# Prevalence, severity and risk factors for mental disorders among sexual and gender minority young people: a systematic review of systematic reviews and meta-analyses

**DOI:** 10.1007/s00787-024-02552-1

**Published:** 2024-08-14

**Authors:** Jonathan O’Shea, Rebecca Jenkins, Dasha Nicholls, James Downs, Lee D. Hudson

**Affiliations:** 1 Population, Policy and Practice Department, Great Ormond Street UCL Institute of Child Health, London, UK; 2 Hull York Medical School, University of York, University Road, Heslington, York UK; 3 Division of Psychiatry, Department of Brain Sciences, Imperial College London, London, UK; 4 Independent Researcher and Expert by Experience, Cardiff, UK

**Keywords:** LGBTQ+, Lesbian, Gay, Bisexual, Transgender, Mental disorder, Depression, Child, Adolescent, Young adult

## Abstract

Evidence suggests heightened prevalence and severity of mental disorders among sexual minority (SM) and gender minority (GM) young people. Several risk factors have been associated with these disparities. A systematic review of systematic reviews and meta-analysis was conducted to provide a comprehensive overview of the literature and to determine the field’s current position. MEDLINE, PsycInfo, Scopus and Web of Science were searched in March 2022 and updated January 2024. Eligibility criteria were systematic reviews or meta-analyses assessing contemporaneous prevalence, severity and/or risk factors of mental disorders among SM or GM young people aged 25 and under. 42 reviews were included, all of which were low quality. The prevalence of depression among SM was 26% (95% CI 21–32%), and among GM was 46% (95% CI 36–56%). Greater depression severity was found among SM compared to heterosexual young people, with a significant albeit small effect size (Hedges’ *g* = 0.38, 95% CI = 0.25 to 0.50); effect sizes were similar when separating by gender. GM also reported greater symptom severity compared to cisgender young people. Other mental disorders were more prevalent compared to those reported in the general population, and of greater severity compared to heterosexual/cisgender young people. Several proximal and distal risk factors were focused upon within the systematic reviews identified. Past systematic reviews consistently indicate a heightened risk of mental disorders among SM and GM young people. Services need to be aware of these disparities and adapt their care accordingly.

**Supplementary Information:**

The online version contains supplementary material available at 10.1007/s00787-024-02552-1.

## Introduction

Mental health is an important issue for sexual minority (SM: individuals who do not exclusively identify as heterosexual) and gender minority (GM: who do not exclusively identify with the sex they were assigned at birth) young people, where unique stressors compared to heterosexual/cisgender peers may intersect with established risk factors experienced by all [1]. Sexual and gender minority (SGM) groups are reported to also experience issues with provision of mental health services, in particular the reinforcement of discrimination and a lack of response to the specific needs of this group [2].

The Minority Stress Model [3], has been influential in explaining disparities experienced by SGM groups. This model considers the role of stressors experienced by sexual and gender minorities (SGM) in the development of mental disorders. These stressors can be distal, which are contextual and encompass the environmental challenges SGM young people can face. They can also be proximal, which encompass the internal processes that occur as a reaction to perceived stigma. More recently, research has examined the unique stressors and consequent mental health problems faced by individuals with intersecting social identities alongside SGM identity [4–6].

Understanding the unique mental health problems faced by SGM young people is vital for a range of stakeholder groups to improve care, in particular policy makers and clinicians. To aid this, a number of systematic reviews (SR) have addressed prevalence and severity of mental disorders as well as risk factors for SGM young people compared to their heterosexual and cisgender peers, These SR have found greater prevalence and severity of depression, generalised anxiety and eating disorders amongst other disorders, with GM young people reported as being particularly vulnerable [7–10]. That said, there are important methodological issues with existing SR. Papers included across reviews are inconsistent; meta-analyses have tended to combine significantly different age groups together [11], and inclusion criteria in some reviews have not been sufficiently rigorous over the definition and measurement of mental disorders [12]. SR assessing risk factors have also tended to focus on one risk factor and/or one mental disorder, rather than providing a comprehensive overview to aid clinicians [13, 14]. As many of the mental disorders discussed in these SR co-occur, this feature of research is ill-matched to the reality.

In an era of multiple, methodologically and content inconsistent SR [15], systematic reviews of systematic reviews offer a unique opportunity for comprehensive and rigorous synthesis of current SR literature [16]. The use of a systematic review of systematic review methodology is well established to synthesise the results of SR assessing health outcomes, particularly when there have been many SR conducted over time [17–19].

We therefore conducted a systematic review of systematic reviews with meta-analyses focusing on (1) prevalence of mental disorders in SM and GM young people; (2) differences in mental disorder severity between SM/GM and heterosexual/cisgender young people and (3) risk factors for mental disorders among SM and GM young people.

## Methods

We used PRISMA guidance as a framework for this review and meta-analysis. Our research team included a researcher with lived experience of both being SGM and having been diagnosed with multiple mental disorders. We searched the databases MEDLINE, PsycInfo, Scopus and Web of Science from 23rd March 2022, with searches updated on 31st January 2024. We searched for published SR and meta-analyses on “mental disorders” and “child/adolescent/young adult” and “LGBTQ+”. groups. Specific search terms for each database are shown in Table S1. We did not use a specific SR filter or search term as we did not want to exclude any potential papers that have not been categorised in this way on the databases.

Inclusion criteria were: (1) SR and/or meta-analyses reporting studies on prevalence of mental disorders within any group of SM and/or GM young people, defining prevalence as reaching on or above the clinical cut-off on a contemporaneous validated mental health tool or contemporaneous clinical diagnoses; (2) SR and/or meta-analyses reporting studies comparing any group of SM/GM with heterosexual/cisgender young people on severity of mental disorders with a contemporaneous validated mental health tool; (3) SR reporting risk factors for a mental disorder and/or its symptoms in any SM or GM group (risk factors defined as moderators, mediators, and associated variables involved in the relationship between SGM identity and contemporaneous mental disorder symptoms and/or diagnosis assessed using validated screening measures).

Exclusion criteria were (1) SR and/or meta-analyses only reporting studies that did not separate SM from GM groups, as we were particularly interested in how SM and GM young people may differ in prevalence of mental disorders; (2) SR and/or meta-analyses only reporting studies where participants over 25 years were combined with young people aged 25 and under; (3) SR and/or meta-analyses only reporting studies assessing mental disorders among participants with pre-existing vulnerabilities (e.g. suicidality, other mental disorders).

Deduplication and screening were conducted manually using EndNote 20. Abstracts, full text screening for inclusion, and bias were assessed independently by two researchers (JO and RJ), with agreement by a third researcher (LH). Quality of included SR was assessed using the Assessing the Methodological Quality of Systematic Reviews 2 (AMSTAR-2) tool [20].

### Data extraction

We extracted data from studies presented within the included SR that showed (1) prevalence rates of mental disorders within SM and GM groups, (2) mean scores on screening tools indicating severity of mental disorder symptoms comparing SM with heterosexual and GM with cisgender groups and (3) risk factors for mental disorders within SM and GM groups. If the SR did not include data on mental disorder prevalence or severity within it, but referred to it qualitatively, we extracted data from the relevant individual studies.

### Meta-analyses and qualitative synthesis

Only pooled prevalence of depression among SM and GM groups could be established in meta-analysis, due to a lack of studies assessing other disorders. Furthermore, only meta-analyses comparing depression severity between SM and heterosexual young people could be conducted, again due to a lack of eligible evidence. These meta-analyses included studies from systematic reviews that (1) reported the number of SM and/or GM participants who indicated clinically significant depression symptoms on a screening tool/reported a contemporaneous diagnosis and (2) included the mean scores and standard deviations/errors on depression screening measures reported by both SM and heterosexual young people. Studies that assessed prevalence of other mental disorders, presented mean scores of depression comparing GM with cisgender young people, and mean scores of other mental disorders comparing SM with heterosexual and GM with cisgender young people were synthesised qualitatively. Individual studies extracted from SR and used for meta-analysis or synthesised qualitatively to determine mental disorder prevalence and severity were assessed for bias using the Newcastle-Ottawa scale (NOS), adapted for use with cross-sectional studies [21]. Each relevant study was assessed by JO.

Meta-analyses were conducted using Stata (v.17) in two parts using random effects models. All studies we found reporting prevalence of depression amongst SM and GM young people were pooled to provide overall estimates with 95% confidence intervals using the metaprop command and applying a Freeman-Tukey arcsine transformation [22]. We assessed publication bias using the Luis Furuya-Kanamori (LFK) index [23]. We also pooled studies reporting results of depression screening tools to generate a standardised mean difference (SMD) between SM and heterosexual young people as Hedges’ *g* with 95% confidence intervals [24]; this was chosen to account for the differing scales used to assess depression. We apriori quantified effect sizes as per Cohen [25] with minimal (< 0.20), small (> 0.20), medium (> 0.50) or large (> 0.80) as is convention. We assessed publication bias using funnel plots and Egger’s test, using the metabias command on Stata [26, 27].

## Results

**Fig. 1:**
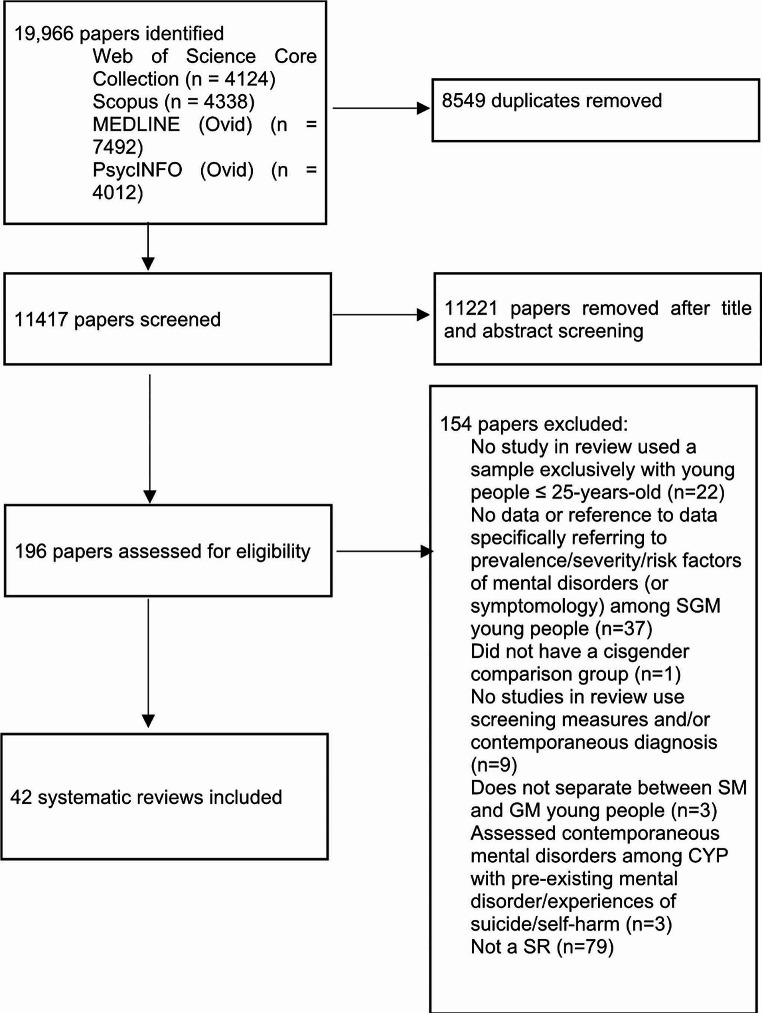
Study selection flowchart

11,417 abstracts were screened, 196 articles were retrieved and assessed in full text, resulting in 42 SR meeting the inclusion criteria (Table 1). A summary of the search strategy can be found in Fig. 1. Here we present findings by (1) prevalence data available for SM young people by mental disorder; (2) SMD between SM and heterosexual groups; (3) risk factors for mental disorders within SM groups; (4) prevalence data available for GM young people by mental disorder; (5) mean differences between GM and cisgender groups and (6) risk factors for mental disorders within GM groups. All studies retrieved from SR for meta-analysis had a NOS score of > = 4, indicating mixed quality research (see Table S2).

### Systematic review summary

All SR were either low or critically low quality using the AMSTAR-2 scale (see Table 1). 91% (38/42) of SR reported eligible studies from North America, specifically the US and/or Canada. 33% (14/42) of SR reported eligible studies from Europe, with all but one of these SR identifying research from Northern or Western Europe. 26% (11/42) of SR reported research from Australia or New Zealand and 12% (5/42) from Asia (specifically China, Thailand and the Philippines). 57% (24/42) of SR synthesised eligible studies with SM only, 31% (13/42) with GM only and 12% (5/42) with both SM and GM. All studies synthesised by the SR was published between 1996 and 2022. All eligible studies were quantitative.

83% (35/42) of the SR included eligible studies which assessed prevalence, severity and/or risk factors for depression, 36% (15/42) assessed generalised anxiety disorder, 14% (6/42) assessed post-traumatic stress disorder and substance use disorders each, 10% (4/42) assessed conduct disorder and eating disorders each, with less than 10% (< 4/42) of SR including research which examined separation anxiety disorder, social anxiety disorder, somatic symptom disorder, oppositional defiant disorder and borderline personality disorder.

**Table 1:** List of included SR.

SR/meta-analysis authors	Countries of eligible included studies within systematic review of systematic reviews	SR/meta-analysis review quality^a^	Number of studies in SR/meta-analysis	Group in eligible studies within SR/meta-analysis^b^	Disorders assessed in eligible studies within SR/meta-analysis
Abreu & Kenny (2018)	1 (USA)	Critically Low	27	SM	Depression
Ancheta, Bruzzeze & Hughes (2021)	2 (New Zealand, USA)	Critically Low	6	SM	Depression
Argyriou, Goldsmith and Rimes (2021)	3 (Netherlands, Sweden, USA)	Low	40	SM	Depression
Baker et al. (2021)	3 (Netherlands, Spain, USA)	Low	20	GM	Depression
Berger, Taba, Marino, Lim & Skinner (2022)	1 (USA)	Low	26	SM	Generalised Anxiety, Depression
Bouris et al. (2010)	3 (Canada, New Zealand, USA)	Critically Low	31	SM	Generalised Anxiety, Depression, Post-Traumatic Stress Disorder
Brown, Porta, Eisenberg, McMorris, & Sieving (2020)	2 (Thailand, USA)	Critically Low	16	GM	Generalised Anxiety, Depression, Post-Traumatic Stress Disorder
Caldarera, Vitiello, Turcich, Bechis & Baietto (2022)	3 (Canada, Netherlands, USA)	Critically Low	9	Both	Separation Anxiety Disorder, Social Anxiety Disorder
Campbell et al. (2024)	1 (USA)	Critically Low	26	GM	Eating Disorders
DeSon & Andover (2023)	2 (Canada, USA)	Critically Low	45	SM	Depression, Alcohol Use Disorder
Dürrbaum & Satler (2020)	3 (Canada, New Zealand, USA)	Critically Low	7	SM	Depression, Post-Traumatic Stress Disorder
Freitas et al. (2017)	1 (USA)	Critically Low	13	SM	Generalised Anxiety, Depression
Frew, Watsford & Walker (2021)	3 (Canada, Netherlands, USA)	Low	15	GM	Generalised Anxiety, Depression
Gilbey, Mahfouda, Ohan, Lin & Perry (2020)	3 (Netherlands, UK, USA)	Critically Low	10	SM	Depression
Hall (2018)	3 (Canada, New Zealand, USA)	Critically Low	35	SM	Depression
Johns et al. (2018)	1 (USA)	Critically Low	21	GM	Depression, Post-Traumatic Stress Disorder
Lekwauwa, Funaro and Doolittle (2022)	1 (USA)	Critically Low	18	Both	Alcohol Use Disorder, Depression
Lucassen, Stasiak, Samra, Frampton & Merry (2017)	5 (Canada, China, New Zealand, UK, USA)	Critically Low	23	SM	Depression
Ludvigsson et al. (2023)	2 (Netherlands, USA)	Low	24	GM	Generalised Anxiety, Depression
Mahon (2021)	1 (USA)	Critically Low	46	SM	Social Anxiety Disorder
Marconi et al. (2023)	3 (Australia, China, USA)	Critically Low	21	GM	Generalised Anxiety, Depression
Marshal et al. (2008)	1 (USA)	Critically Low	18	SM	Substance Use Disorders
Marshal et al. (2011)	2 (Canada, USA)	Low	24	SM	Depression
McCann & Brown (2019)	2 (Canada, USA)	Critically Low	14	SM	Depression
McCann, Donohue and Timmins (2020)	1 (USA)	Critically Low	9	SM	Depression
McDonald (2018)	2 (Canada, USA)	Critically Low	10	Both	Conduct Disorder, Depression
Meneguzzo et al. (2018)	1 (Norway)	Critically Low	45	SM	Eating Disorders
Mezzalira et al. (2022)	4 (Canada, China, New Zealand, USA)	Critically Low	33	GM	Generalised Anxiety, Conduct Disorder, Depression, Separation Anxiety Disorder, Somatic Symptom Disorder
Millet, Longworth & Arcelus (2017)	3 (Canada, Netherlands, USA)	Critically Low	25	GM	Generalised Anxiety
Newcomb and Mustanski (2010)	1 (USA)	Critically Low	31	SM	Generalised Anxiety, Depression
Pinna et al. (2022)	2 (Canada, UK)	Critically Low	165	GM	Generalised Anxiety, Depression
Plöderl & Tremblay (2015)	4 (New Zealand, the Philippines, UK, USA)	Critically Low	199	SM	Alcohol Use Disorder, Generalised Anxiety, Borderline Personality Disorder, Conduct Disorder, Depression, Oppositional Defiant Disorder
Pompili et al. (2014)	1 (USA)	Critically Low	19	Both	Conduct Disorder, Depression, Eating Disorders, Post-Traumatic Stress Disorder
Shokoohi et al. (2022)	2 (Australia, USA)	Critically Low	105	SM	Alcohol Use Disorder
Singh, Dandona, Sharma, & Zaidi (2023)	1 (USA)	Critically Low	42	SM	Generalised Anxiety, Depression, Substance Use Disorder
Tankersley, Grafsky, Dike and Jones (2021)	2 (Canada, USA, inc. Puerto Rico)	Low	44	GM	Generalised Anxiety, Depression, Post-Traumatic Stress Disorder, Separation Anxiety Disorder
Tsarna et al. (2022)	1 (Greece)	Critically Low	20	GM	Generalised Anxiety, Depression
Valentine and Shipherd (2018)	1 (USA)	Critically Low	77	GM	Depression
Vrangalova and Savin-Williams (2014)	1 (Norway)	Critically Low	60	SM	Depression, Eating Disorders
Williams et al. (2023)	1 (USA)	Low	22	Both	Depression
Wittgens et al. (2022)	2 (Iceland, New Zealand)	Critically Low	26	SM	Depression
Xu et al. (2023)	2 (Australia, USA)	Critically Low	23	SM	Depression

^a^ assessed with the AMSTAR 2 scale, ^b^ SM = sexual minorities, GM = gender minorities

### Prevalence of mental disorders among sexual minorities

#### Depression

**Fig. 2:**
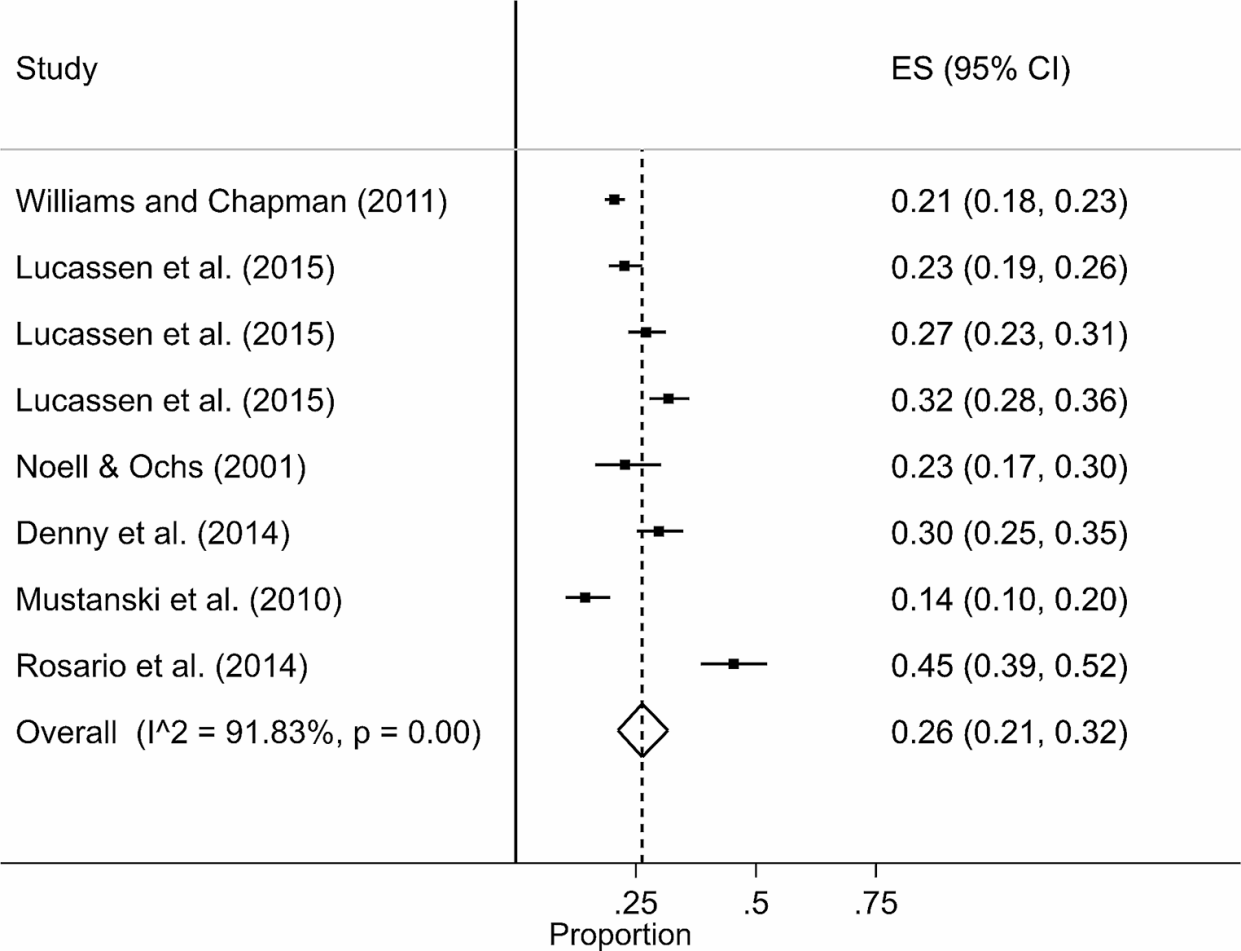
Forest plot for prevalence of SM young people with depression, with pooled estimates of overall prevalence with 95% CIs with dotted line also showing the pooled overall weighted mean estimate of prevalence = 26%; and ES = effect size)

Six individual studies [28–33] from five SR [11, 12, 34–36] were included in the meta-analysis examining prevalence of depression. The study by Lucassen et al. (2015) collected data over three timepoints with different participants at each timepoint, hence it has been included three times within the meta-analysis. All these studies used community or convenience samples. Due to lack of data, we could not split the meta-analyses by gender, meaning we only meta-analysed studies that included prevalence data mixing males and females. The overall pooled estimate of prevalence was 26% (95% CI 21–32%) (Fig. 2). The LFK index was 1.89, indicating minor asymmetry and thus a moderate risk of bias.

In two studies from three SR [12, 34, 35] assessing depression among SM females separately (i.e., not analysed), we identified prevalence rates of between 25.9 and 38.6% [29, 30]. For SM males, we identified prevalence rates of between 19.2 and 24.2% [29, 30].

#### Other disorders

We identified two studies from three SR [36–38] examining contemporaneous prevalence of eating disorders, with one study reporting a 1.3% and 0.7% prevalence of anorexia nervosa and bulimia nervosa respectively among lesbian and gay young people using diagnostic interviews [32]. Furthermore, we identified research reporting bulimia nervosa prevalence of between 5.6 and 21.6% when using a self-report measure [39].

We identified three studies examining prevalence for substance use disorders from two SR [40, 41], with homeless lesbian, gay and bisexual young people reporting a 52.4% prevalence of alcohol use disorder, and 47.6% prevalence of substance use disorder [42]. Among a community sample of young adult SM, proportions of participants above the threshold for alcohol use disorder were between 59 and 78%, with the highest prevalence among SM females [43]. On the other hand, research has suggested alcohol use disorder prevalence are much lower among a sample inclusive of adolescents, with prevalence of between seven and nine percent [44].

We identified one study from one SR [36] examining prevalence of conduct disorders and post-traumatic stress disorder, with prevalence of conduct disorder being 18.5% among lesbian and gay young people and 12.9% among bisexual young people, and prevalence of post-traumatic stress disorder being 11.3% of lesbian and gay young people and 7.1% of bisexual young people [32].

### Comparisons of mean mental disorder scores in sexual minority versus heterosexual young people

#### Depression

**Fig. 3:**
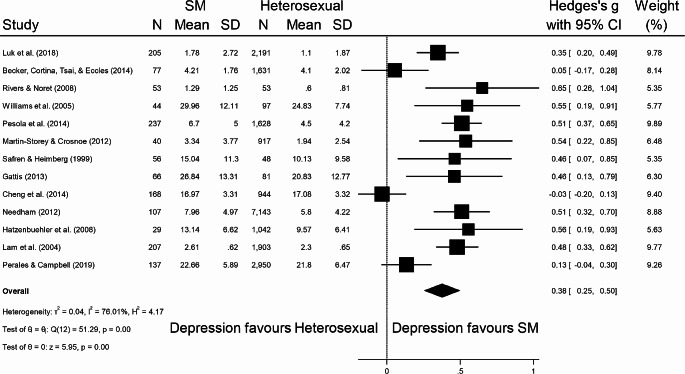
Standardised mean difference meta-analysis for SM young people with depression with overall effect size (Hedges’ *g*) and 95% confidence intervals

**Fig. 4:**
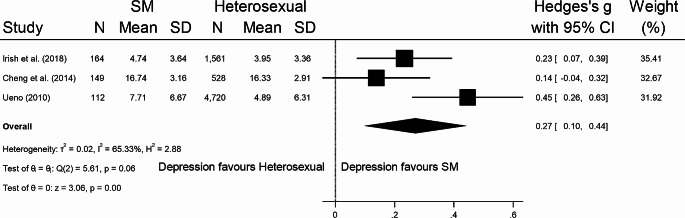
Standardised mean difference meta-analysis for SM male young people with depression with overall effect size (Hedges’ *g*) and 95% confidence intervals

**Fig. 5:**
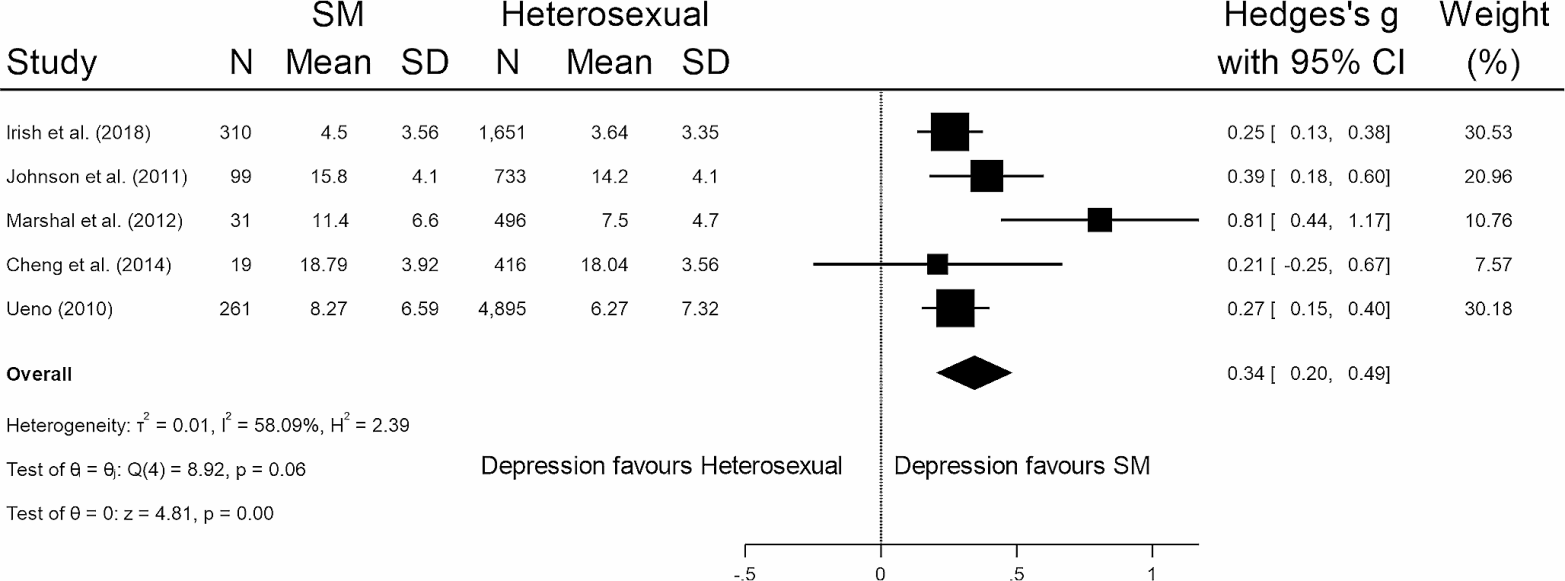
Standardised mean difference meta-analysis for SM female young people with depression with overall effect size (Hedges’ *g*) and 95% confidence intervals

Seventeen studies [45–61] from seven SR [12, 34, 35, 62–65] provided data that enabled them to be included in meta-analysis. Overall, SM young people possessed more severe depressive symptoms compared to heterosexual young people, with a significant albeit small effect size (Hedges’ *g* = 0.38, 95% CI = 0.25 to 0.50, *p* < .001) (Fig. 3); these results were consistent for males (Hedges’ *g* = 0.27, 95% CI = 0.10 to 0.44, *p* < .001) (Fig. 4) and females (Hedges’ *g* = 0.34, 95% CI = 0.20 to 0.49, *p* < .001) (Fig. 5). Eggers’ test and inspection of funnel plots indicated the studies within the mixed meta-analysis did not have significant publication bias (*z* = 1.48, *p* = .14).

We identified three studies [33, 66, 67] reviewed by three SR [11, 12, 35] presenting separate means for lesbian and/or gay and bisexual young people, finding elevated depression symptoms among both groups compared to heterosexual young people. One study identified by one SR [12] did not report standard deviations; however, it did find significantly higher depressive symptoms among SM compared to heterosexual young people when comparing most subgroups [68] (see Table S3 for details on these studies).

#### Anxiety

We identified four studies [54, 60, 69, 70] from two SR [12, 13] examining differences in both mean generalised and social anxiety symptoms, finding significantly more severe symptoms in SM compared to heterosexual young people in five of the six scales used (see Table S3).

#### Other disorders

We identified two studies [60, 69] from one SR [12] that looked at oppositional defiant disorder, conduct disorder and borderline personality disorder/emotionally unstable personality disorder symptom scores among SM girls, which were significantly higher among this group compared to heterosexual girls (see Table S3).

We identified two studies from two SR comparing average substance use disorder symptoms between SM and heterosexual young people [12, 40]: one study described gender differences, finding female SM young people were more likely to report more severe tobacco dependence compared to heterosexual females, although SM male young people had the opposite results [71]. However, mixed evidence was found for significant differences in alcohol use disorder symptoms between lesbian, gay and bisexual and heterosexual young people [49] (see Table S3).

### Risk factors for mental disorders amongst sexual minority young people

Table 2 presents a summary of the risk factors for mental disorders experienced by SM young people, separated by proximal and distal factors.

**Table 2:** Proximal and distal risk factors for mental disorders among SM young people

Proximal factors	Distal factors
Maladaptive coping mechanisms/emotion regulation	Lack of closeness with and hostility from family members
Internalised homophobia/discomfort with sexual identity	Lack of support from friends
Lack of openness about sexual orientation/stress associated with ‘coming out’	Bullying victimisation/discrimination/harassment/microaggressions
	Lack of school support
Vulnerable groups (asexual and bisexual young people, adolescents, females)	Hostility from religious groups
Early frequent sexual activity	Stressful life events
	Traumas experienced in childhood and adolescence
Internal religious conflicts	
Unmet medical needs	

### Comfort with sexual orientation

Thirteen studies [72–84] from four SR mostly identified positive associations between internalised homophobia and depression, generalised anxiety and post-traumatic stress disorder [85–88]. Internalised homophobia can be defined as incorporating negative societal attitudes towards SM into one’s own belief system [89] and can involve a conflict between one’s sexual attractions and the need to fit into a heteronormative society [90]. Four studies [70, 81, 83, 84] from two SR indicated discomfort, concealment of and interpersonal struggles with one’s SM identity were associated with depression and social anxiety disorder, although evidence for this was mixed [13, 86].

Conversely, two studies [83, 91] from one SR indicated positive attitudes towards homosexuality and sexual identity integration (i.e. internal and external embracement of one’s sexual identity) were negatively associated with depression although the latter association was only significant for consistently high levels of integration [86]. Comfort with sexual identity was indicated by one study [70] from one SR to be negatively associated with social anxiety disorder, although evidence for this was mixed [13]. We identified one study [83] from one SR noting potential gender differences in this effect, with evidence to suggest the relationship between comfort with homosexuality with depression was significant for males only [86]. Furthermore, we found one study [92] from one SR that reported evidence suggesting SM identity salience (i.e., importance in incorporating sexual orientation into one’s overall identity) was associated with reduced generalised anxiety disorder symptoms when faced with distal stressors, but greater anxiety when facing proximal stressors [93].

### Coping mechanisms and behaviours

Four studies [54, 74, 94, 95] from four SR indicated protective factors of depression, including mastery (i.e. perceived control over one’s life), emotional awareness, perceived competence to deal with difficult situations, resiliency and feelings of mattering and belonging; conversely, the same reviews reported positive associations between depression and anxious personality traits, rumination and feelings of burdensomeness and strain, along with emotion regulation deficits [86, 93, 96, 97]. Five studies [83, 94, 98–100] from two SR indicated self-esteem was negatively associated with depression, although findings were mixed [86, 96]. Furthermore, one study [70] from one SR indicated lower self-esteem was associated with symptoms of social anxiety disorder [13].

Two studies [51, 101] from two SR described research indicating the role of maladaptive coping strategies. General coping strategies (e.g. self-blame) partly mediated the relationship between internalised homophobia and both depression and anxiety among SM female young people [93]. Levels of coping through acceptance partly attenuated the relationship between sexual orientation and depression [96]. However, coping strategies specific to sexual orientation (e.g., negotiating one’s own internalised homophobia) were not significantly associated with depression nor anxiety [93].

### Sociodemographics

One study [102] from one SR indicated Asian SM young people were less likely to report anxious symptoms compared to their White peers [103]. Conversely, we found one study [104] from one SR indicating no differences in average social anxiety disorder symptoms across ethnic groups among SM females [13]. An intersectional risk factor was noted by one study [105] from one SR, which indicated more severe depressive symptoms were reported by Black SM young people who had experienced intersectional microaggressions (i.e. discriminatory acts based on both racial and sexual identities) [106]. One study [107] from one SR indicated the gap between SM and heterosexual girls and bisexual young people of any gender in depressive symptoms widened between adolescence and young adulthood, suggesting an age effect [62]. Two studies [70, 108] from two SR indicated gender nonconformity was positively associated with both depression and social anxiety disorder, although for the latter the relationship was significant for only one of four scales used to assess social anxiety [13, 86]. Looking at subgroup differences, one study [109] from one SR indicated asexual young people experienced more severe symptoms of depression compared to both bisexual and gay/lesbian young people [65]. Additionally, one study [74] from one SR indicated bisexual females were at particular risk of depressive symptoms compared to gay men [88].

### Openness about sexual orientation and engagement with SGM community

Two studies [81, 95] reported in two SR indicated positive associations between stress linked to ‘coming out’ with depression [85, 86]. One study [70] from one SR indicated a lack of openness was associated with social anxiety disorder [13]. Conversely, we identified three studies [73, 83, 95] from one SR reporting others’ knowledge of sexual orientation was negatively associated with depression, although one such study [83] indicated this relationship was only significant for males [86]; furthermore, one study [110] from the same SR indicated no significant association between outness and depression.

One study [91] from one SR indicated a negative relationship between symptoms of depression and sexual identity integration, which included involvement in SGM-related activities, positive attitudes towards SM identity, and comfort with and disclosure of one’s sexual identity to others; however, this association was only significant for consistently high levels of integration within a period of twelve months [86]. Furthermore, one study [83] from the same SR indicated no significant association between involvement in SGM nightlife activities and depression [86].

### Stressful life events

Two studies [51, 94] reported by one SR indicated experiencing fewer positive and more negative life events individually attenuated the relationship between sexual orientation and depression [96]. Additionally, one study [111] reported by one SR indicated stressful life events were directly associated with depressive symptoms, although evidence for this was mixed [86].

### Non-sexual orientation-specific family factors

One study [112] from one SR indicated family socioeconomic status was negatively associated with depression [86]. Furthermore, three studies [45, 94, 113] from two SR indicated family satisfaction, support and closeness with parents were mediators in the relationship between sexual orientation and depression, with support and closeness having a stronger mediating relationship among girls [93, 96]. Six studies [73, 82, 91, 111, 113, 114] from three SR also indicated negative associations between family support, engagement in activities with family and family closeness with depression, although mixed evidence was found for support [86, 93, 115]. One study [116] from one SR indicated among bisexual and mostly heterosexual young people, lower attachment security to a maternal figure, but not maternal affection, attenuated the relationship between sexual orientation and depressive symptoms; for lesbian and gay young people, insecure maternal attachment fully attenuated this relationship [96].

### Sexual orientation-specific family factors

One study [117] from one SR indicated a positive association between depression and mothers’ knowledge of sexual orientation, but no significant relationship with father’s knowledge; further, one study [118] from the same SR indicated the relationship between outness to family and depression was not significant [86]. In addition, one study [112] from two SR indicated acceptance by family members was negatively associated with symptoms of depression [36, 86]. Furthermore, we found one study [119] from one SR suggesting acceptance by mothers moderated the association between same-sex attraction and symptoms of social anxiety [120]. However, one study [82] reported in two SR indicated potential sociodemographic differences, finding no significant associations between specific support for sexual orientation and depression symptoms among bisexual young people [86, 115].

On the contrary, one study [121] from one SR indicated young people whose parents used homophobic/transphobic slurs or who discouraged gender atypicality (i.e. not conforming to the stereotypical traits of their birth sex) were more likely to report symptoms of post-traumatic stress disorder [115]. Four studies [81, 107, 117, 122] from three SR indicated rejection by family members both mediated the relationship between sexual orientation and depressive symptoms and was associated with both anxiety and depression, particularly if both parents rejected the young person’s sexual orientation [86, 96, 115]; however, one such study [107] reported the mediating effect of parental rejection was not significant for gay and bisexual males, with another [123] reporting the association between paternal rejection and depression was not significant [86, 96]. Furthermore, one study [117] from one SR indicated stress regarding coming out to parents was not significantly associated with depression [86]. One study [124] from one SR indicated low social acceptance and perceived care by parents partly attenuated the relationship between sexual orientation and depression [96]. Two studies [77, 111] from two SR reported positive associations between experiences of homelessness and depressive symptoms, although this evidence was mixed [63, 86].

### Peer and community support

Seven studies [51, 82, 91, 99, 111, 114, 118] from three SR indicated contact with friends and general social support offered by friends and their community were generally identified as being negatively associated with depressive symptoms or attenuated the relationship between sexual orientation and depressive symptoms; however, no association was found for sexual orientation specific support [86, 96, 115]. Additionally, one study [125] from one SR indicated no significant associations between online support on social media and both depression and anxiety [126]. One study [124] from one SR indicated lower social support by friends, along with the suicide of a friend, partly attenuated the relationship between sexual orientation and depression [96]. Another study [127] from the same SR indicated feelings of social isolation fully attenuated the relationship between sexual orientation and depression among males [96]. However, two studies [117, 118] from one SR indicated no significant associations between depressive symptoms and both being out to friends and the stress of coming out to friends [86]. Furthermore, one study [45] from one SR indicated no mediating influence of peer support on the relationship between sexual orientation and depression [96]. On the other hand, two studies [51, 128] from two SR indicated social support satisfaction was both negatively associated with social anxiety disorder, and partly attenuated the relationship between sexual orientation and depression [13, 96]. Additionally, three studies involving the same sample [91, 99, 111] from one SR indicated positive associations between the number of negative social interactions and depression, although these findings were mixed [86]. However, one study [129] from the same SR indicated no significant associations between both SGM community size and community climate with depression [86].

### Victimisation

We identified eighteen studies [45, 50, 72, 81, 94, 95, 107, 108, 110, 114, 121, 123, 130–135] from seven SR that described evidence to suggest victimisation, discrimination, microaggressions, harassment and abuse of various forms, including physical, verbal, cyber and sexual were associated with or a mediating factor in depression, alcohol use disorder and/or post-traumatic stress disorder [14, 85, 86, 96, 97, 106, 115]. Two studies [110, 114] from one SR with ethnic minority samples indicated associations between discrimination based on ethnicity and sexual orientation with depression, although mixed evidence was found for the latter [86]. Furthermore, one study [124] from one SR indicated the relationship between sexual orientation and depression was attenuated by witnessing victimisation, specifically a shooting or stabbing [96]. However, one further study [130] from the same SR reported evidence indicating the mediating effect of victimisation on the relationship between sexual orientation and depression reduced among twin pairs when accounting for unmeasured family factors, suggesting an interaction between family factors and victimisation in determining mental disorder outcomes [96]. We also identified one study [50] from this SR indicating interactions between various stressors: both a negative perception of the school environment and a lowered sense of self-concept partly mediated the relationship between sexual orientation related harassment and depression [96].

### Sexual & romantic activity

We identified two studies [73, 117] from one SR indicating positive associations between depression and the number of same-sex sexual partners and worrying about one’s sex life, along with negative associations with same-sex sexual contact and age in which young people initiated sexual behaviour [86]. However, one study [83] from one SR indicated a negative relationship between the number of unprotected oral sex experiences and depression among SM male but not female young people; this and another study [117] from the same SR indicated the associations between depression and experiences of anal and vaginal sex, nor the number of overall sex episodes and worries about HIV/AIDS, did not reach significance [86]. Mixed results from one study [128] were reported by one SR for the relationship between condomless anal sex and social anxiety disorder [13]. Furthermore, one study [94] from one SR indicated sexual exploration attitudes did not mediate the relationship between sexual orientation and depression [96]. One study [136] from one SR indicated homeless men with depression who have sex with men were over twice as likely to report trading sex compared to those without depression [63]. An examination on the impact of romantic partners has been covered less, with two studies [73, 118] from one SR suggesting that having a same or opposite-sex romantic partner was negatively associated with depression, although evidence for this was mixed [86].

### School support

Two studies [124, 137] from one SR indicated lower social support and acceptance from teachers, along with feelings of institutional betrayal, partly attenuated the relationship between sexual identity and depressive symptoms [96]. Conversely, two studies [31, 102] from one SR indicated a positive school climate (i.e., a school environment in which SM young people felt supported and safe) was negatively associated with symptoms of depression, although one such study [31] suggested school climate moderated levels of depressive symptoms for male SM only [103]. Furthermore, one study [129] from one SR indicated school climate was not associated with depression amongst university students [86].

### Religion

One study [98] from one SR indicated young people who had positive feelings about God were less likely to report depressive symptoms and vice versa; however, evidence for this was mixed [86]. Additionally, one study [138] from one SR indicated SM young people educated in religious schools had significantly more severe alcohol use disorder symptoms on average compared to SM young people who were not [139]. One study [98] from one SR indicated young people who had a preoccupation with sin experienced greater depression symptoms, although evidence for this was mixed [86]. However, three studies [74, 98, 117] from two SR reported depression was not associated with feeling religion was beneficial, experiencing anxiety over religious beliefs about homosexuality, experiencing comfort from religion in accepting one’s SM identity, nor strength of religious faith [86, 115]. Furthermore, one study [74] from one SR indicated the association between religiosity and depression was not significant amongst a sample of Black lesbian, gay and bisexual young people [93]. Two studies [81, 98] from two SR reported young people who were part of an SGM-inclusive religious environment were less likely to report depressive symptoms, although evidence for this was mixed [86, 140]. Conversely, one study [98] from one SR indicated experiencing stressors from religious groups was positively associated with depressive symptoms [86].

### Online engagement

We identified one study [141] from one SR which indicated increased time engaged in online activities, including social media, mediated the relationship between sexual orientation and depression; however, this was a mediator for bisexual but not gay, lesbian or questioning young people [96]. One study [125] from one SR indicated no significant relationship between use of social media for support and anxious and depressive symptoms; conversely, learning about one’s sexuality through social media significantly reduced generalised anxiety symptoms [126].

### Unmet medical needs

One study [45] from one SR reported unmet general medical needs mediated the relationship between sexual orientation and depression [96].

### Legislation

One study [92] from one SR we identified indicated same-sex marriage accessibility and the presence of work discrimination rules were not associated with generalised anxiety symptoms [93].

### Past experiences

The results of two studies [100, 108] from one SR indicated hiding one’s sexual orientation, along with victimisation based on sexual orientation, at school was associated with depression among SM young adults [86]. Additionally, two studies [111, 129] from one SR indicated childhood trauma, including sexual abuse, was positively associated with depressive symptoms; however, statistical evidence for this was mixed [86]. Two studies [100, 129] from one SR indicated both the presence of a gender-sexuality alliance (i.e. organised social clubs in which SGM young people and allies can socialise and support one another) in school and being ‘out’ at school were negatively associated with depression among young adults [86]. On the other hand, one study [112] from one SR indicated neither family religiosity nor religious affiliation in childhood were found to be associated with depression among young adults [86].

### Prevalence of mental disorders among gender minorities

#### Depression

**Fig. 6:**
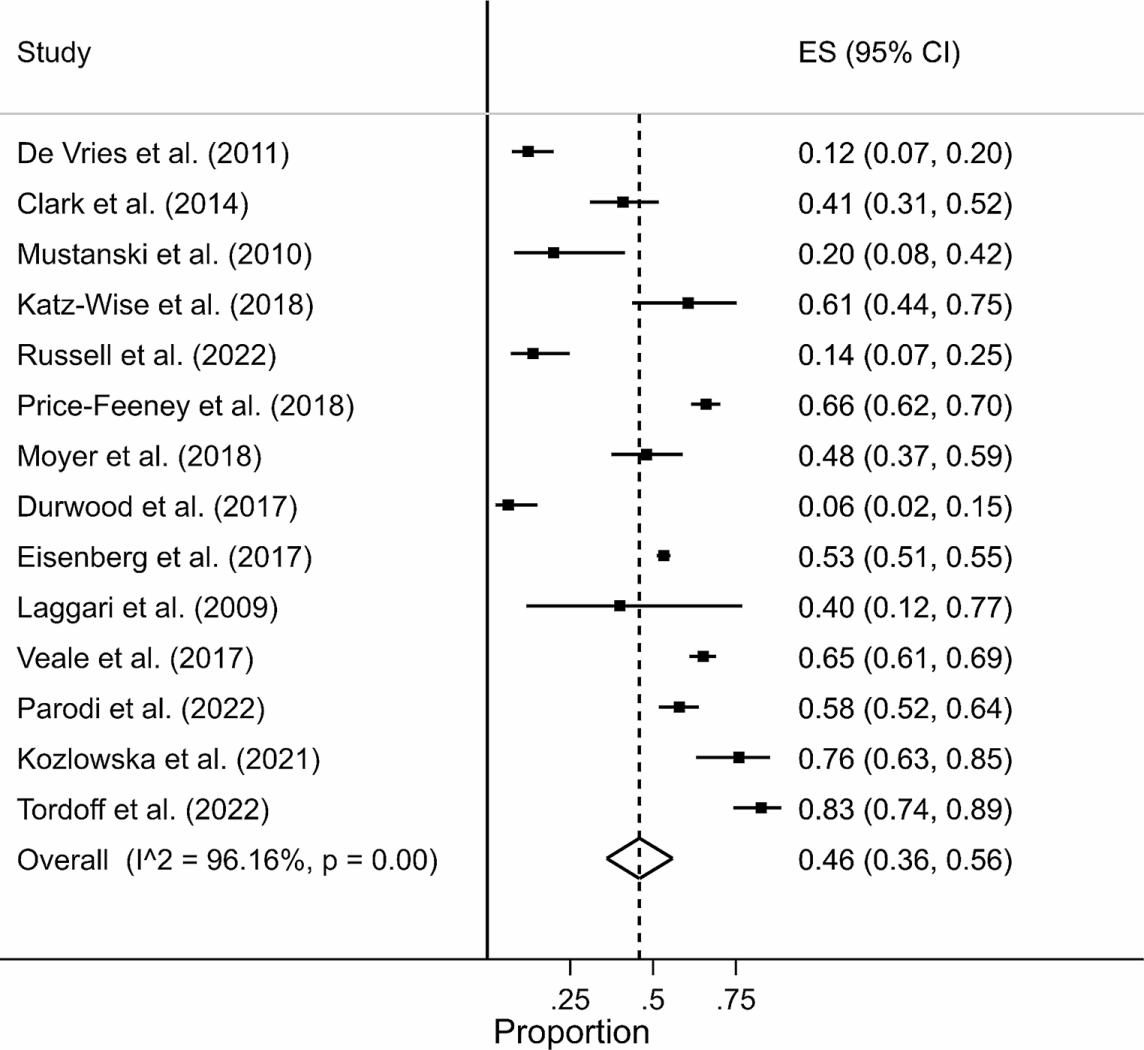
Forest plot for prevalence of GM young people with depression, with pooled estimates of overall prevalence with 95% CIs. The dotted line indicates the pooled overall weighted mean estimate of prevalence = 46%; and ES = effect size)

We analysed fourteen studies [32, 142–154] from seven SR [36, 139, 155–159] eligible for the meta-analysis which reported prevalence of depression among GM young people. There were not enough studies to separate by either assigned sex or identified gender. Most studies consisted of convenience samples from SGM organisations. The overall pooled estimate of prevalence was 46% (95% CI 36–56%) (Fig. 6). Heterogeneity was high (I^2^ = 96%). The LFK index was − 2.00, indicating minor asymmetry and thus a moderate risk of bias.

#### Other disorders

We identified one study from one SR [36] that looked at prevalence rates of post-traumatic stress disorder and conduct disorder respectively, with post-traumatic stress disorder prevalence of 10% and conduct disorder prevalence of 15% among GM young people [32]. The same study found no evidence of eating disorders. Seven studies from four SR [155, 156, 158, 159] found anxiety disorder prevalence of between 13 and 85% [142, 147, 148, 150, 153, 154, 160]. Additionally, we identified one study synthesised in two SR [120, 156], which found a prevalence of 5.3% for separation anxiety disorder using DSM-IV conservative criterion [161]. We identified one study from one SR [156] conducted during the COVID-19 pandemic, which found a post-traumatic stress disorder prevalence of 46% and anxiety prevalence of 70% [152].

### Comparisons of mean mental disorder scores between gender minority and cisgender young people

We identified five studies [145, 148, 162–164] from four SR [64, 155, 156, 165] finding GM young people possessed more severe symptoms of anxiety, depression, conduct disorder and somatic symptom disorder compared to cisgender young people, although among the studies identified these differences did not reach statistical significance or significance was not reported. Furthermore, we identified one study from one SR [158] that found intersex young people with Mayer-Rokitansky-Küster-Hauser syndrome, a congenital disorder characterised by aplasia of the vagina and uterus among individuals assigned female at birth, experienced more severe depressive and anxious symptoms compared to a control group of young people, although the statistical significance of the difference between the mean scores was not reported [150] (see Table S4 for mean scores among each group).

### Risk factors for mental disorders amongst gender minority young people

Table 3 presents a summary of the risk factors for mental disorders experienced by GM young people, separated by proximal and distal factors.

**Table 3:** Proximal and distal risk factors for mental disorders among GM young people

Proximal factors	Distal factors
Negative coping mechanisms	Lack of acceptance/rejection from family
Internalised transphobia	Lack of support from peers/schools
Low appearance congruence	Victimisation/discrimination
Detectable HIV viral load	Living in suburban area
Vulnerable groups (non-binary, older adolescents, assigned male at birth)	
Drug use	
Trading sex	

### Coping mechanisms

Two studies [166, 167] from three SR indicating negative associations between resiliency and personal mastery with post-traumatic stress disorder; the latter was also associated with depression [168–170]. Conversely, two studies [161, 166] from two SR indicated a positive relationship between emotion-oriented coping methods, along with behavioural and emotional problems, and depression, post-traumatic stress disorder, and separation anxiety symptoms [156, 170]. However, one study [166] from one SR indicated self-esteem was not significantly associated with depressive nor trauma symptoms [169].

### Response to gender identity

Two studies [171, 172] from three SR indicating positive associations between internalised transphobia and both depression and anxiety disorders [88, 156, 170]. Furthermore, one study [171] reported in the same two SR indicated depression was negatively associated with appearance congruence, referring to the level of alignment one’s physical traits have with their gender identity [156, 170].

### Diagnostic and medical factors

One study [173] from one SR indicated GM assigned male at birth who met complete criteria for gender identity disorder were more likely to report separation anxiety disorder compared to those who did not, although this was for a liberal but not conservative definition of separation anxiety disorder [120]. We identified five studies [142, 174–177] from two SR which reported comparatively mixed results for the relationship between hormone therapy and depression, with most reporting improvement but others reporting no difference [178, 179]. One study [180] from one SR indicated gender affirming care did not lessen symptoms of eating disorders, namely binge eating disorder and avoidant-restrictive food intake disorder (ARFID) [181]. Two studies [174, 182] from two SR indicated mixed changes in generalised anxiety symptoms post sex-reassignment surgery; one such study [174] indicated no significant effect on depression [165, 178]. One study [183] from one SR indicated amongst HIV-positive GM female young people, those with a detectable viral load were more likely to screen positive for depression [170].

### Sociodemographics

Two studies [152, 184] from two SR indicated non-binary young people reported greater severity of depression and anxiety compared to binary transgender young people before and during the COVID-19 pandemic [156, 157]. One study [185] from one SR indicated older adolescents reported more severe depressive symptoms compared to their younger counterparts [170]. Furthermore, one study [161] from one SR indicated young people assigned male at birth, but not those assigned female at birth, were at greater risk of experiencing separation anxiety disorder compared to the general population [120].

### Substance use

We identified one study [186] from one SR reporting evidence to suggest GM young people who reported drug use experienced nearly twice the odds of reporting symptoms of post-traumatic stress disorder compared to young people who did not [170].

### Sexual activity and health

We identified one study [187] from one SR suggesting GM young people who reported trading sex had more severe anxious and depressive symptoms [156]. Conversely, one study [188] from one SR indicated anxiety and depression was not associated with any diagnosis of a sexually transmitted infection nor condomless anal and/or vaginal sex within the past six months [170].

### Family factors

One study [161] from one SR indicated separation anxiety was higher among young people assigned male at birth whose parents were not living together [170]. Furthermore, one study [189] from the same SR indicated young people with feelings of connectedness to one’s parents and wider family were less likely to report depression [170]. One study [144] reported in two SR indicated depression and anxiety symptoms were negatively associated with young people’s quality of communication and satisfaction with family [156, 168]. Three studies [167, 190, 191] from five SR described research suggesting family support and affection was negatively associated with depressive symptoms, with the latter also being associated with post-traumatic stress disorder [156, 168–170, 192]. Additionally, one study [167] from two SR suggested acceptance by family was negatively associated with symptoms of post-traumatic stress disorder but not depression [168, 170]. Conversely, one study [193] from one SR indicated young people who were rejected by their family due to their gender identity were more likely to report symptoms of depression [168].

### Friend and school-specific factors

Two studies [166, 190] from three SR indicated social support from peers and schools was negatively associated with anxious and depressive symptoms, although it was not associated with trauma symptoms; one such study [190] also indicated peer and school support moderated the relationship between GM-related victimisation and anxious and depressive symptoms [156, 169, 170]. Additionally, one study [189] from one SR indicated young people who viewed their school as more safe were less likely to experience depression symptoms [170]. Conversely, one study [161] reported in one SR indicated poor peer relations were related to separation anxiety symptoms [156].

### Victimisation

We identified one study [167] from two SR reporting discrimination based on GM identity was associated with both post-traumatic stress disorder and depression [170, 192]. Additionally, one study [146] from one SR indicated gender-nonconforming young people who were victimised based on their religion were more likely to report depressive symptoms compared to those who did not, whilst those victimised based on their ethnicity were less likely to report depressive symptoms [139].

### Wider community response

One study [194] from one SR indicated higher depression rates amongst GM young people who lived in suburban areas compared to GM young people from rural or urban areas; one study [189] from the same SR indicated connectedness to adults within the community was negatively associated with depression [170]. One further study [195] from the same SR indicated young people who were called by their chosen name in a greater number of contexts were less likely to report depressive symptoms [170].

## Discussion

We believe this to be the first systematic review of systematic reviews on mental disorders in SM and GM young people, bringing together mental disorder prevalence, severity and risk factors. As these SR and meta-analyses have focused on a specific disorder or set of risk factors within a particular domain, we have aimed to provide a comprehensive synthesis of the prevalence and severity of multiple disorders and risk factors reflecting the interacting environments a SGM young person exists within. Depression was the most reported mental disorder in SR. In meta-analysis we identified a pooled prevalence for depression of 26% among SM young people and 46% among GM young people. For comparison, these rates were lower compared to national estimates of depressive symptom prevalence of 63% and 74% among SM and GM young people [196, 197], although national surveys tend to use single-item measures of depression. Nevertheless, rates remained higher compared to background populations of young people, with SR of research conducted in Europe and North America indicated depression prevalence ranging between 16 and 20% [198, 199]. We also found a significant small to medium effect size (0.4) for increased depression symptom severity scores in SM young people compared to heterosexual young people when pooled in meta-analyses. Whilst we were unable to find sufficient studies identified by the various SR for comparison with other disorders, we did find a range of reported studies which consistently indicated higher prevalence and symptom severity scores for several disorders in SM/GM compared to heterosexual/cisgender young people, including anxiety, post-traumatic stress disorder and eating disorders.

Using the minority stress model as a framework, we have brought together evidence reported in SR indicating risk factors for mental disorders among SGM young people. Factors related to the family were frequently identified, focusing upon the negative impacts of family rejection based upon the young person’s sexual orientation or gender identity. Victimisation and harassment were also noted frequently, particularly for SM. SR also focused upon proximal factors, including one’s level of resilience and the impact of negative coping methods including internalised homophobia and transphobia. Protective factors were frequently noted, in particular family and peer support. Many of the factors indicated by this systematic review of systematic reviews are consistent across the SR, which as previously mentioned have tended to focus on these factors individually and mixed age groups [200, 201]; this systematic review of systematic reviews therefore provides robust evidence for the importance of these factors in determining mental disorder outcomes among SGM young people. It has also determined factors that have received limited attention. For example, there is a lack of evidence on the role of factors outside the young person’s immediate environment: with the passing of legislation impacting SGM people in multiple countries within recent years in both potentially positive (e.g. anti-discrimination laws) and negative ways (e.g. restrictions to gender-affirming care), it will be important to understand both the direct and indirect impacts of these, particularly among young people beginning to develop their sexual and gender identity amidst these changes.

This systematic review of systematic reviews has several strengths and limitations. Using this method has allowed us to synthesise the heterogenous review literature that exists within this field, providing researchers with a comprehensive reflection on what has and has not been focused upon. The meta-analyses also provide robust statistical evidence for the prevalence of depression among SM and GM young people and severity of depression among SM, as all studies used contemporaneous screening measures or diagnostic tools for specific mental disorders and only included young people aged 25 and under. This has built upon previous meta-analyses in this area that have not fulfilled these criteria. We included a researcher with lived experience to collaborate in the design, interpretation and reporting of the study. Separating SM and GM has allowed us to indicate the unique prevalence and severity of mental disorders among each group. Our findings suggest the latter group are more likely to experience significant depressive symptoms, which can indicate to clinicians and researchers that this group should be prioritised. Search terms were agreed a priori, were wide-ranging to capture as many relevant reviews as possible, and multiple databases were searched using two researchers. We used established bias assessment tools for both SR and retrieved individual studies used in meta-analysis. However, most of the SR found were of low quality, principally because of a lack of investigation into the causes of heterogeneity. To address this, we also further bias assessed studies retrieved from SR. NOS scores for these studies were generally high, with common issues being the sampling method used and a lack of detail on non-respondents.

Our findings are also limited by the cross-sectional nature of studies found for prevalence and severity scores. Most studies were convenience samples of small sizes which adds bias in terms of representation. Furthermore, as sexual and gender identity can be fluid for some young people [202, 203], it may be that SGM young people identified by the studies within each SR may not identify as such in the future. The reverse is possible, in that those identifying as heterosexual/cisgender may in the future identify as SGM. To address these challenges and move the research field forward, addressing the methodological limitations requires larger community-based studies with longitudinal follow up across all SGM groups, being inclusive of those with identities other than lesbian, gay, bisexual and transgender (i.e., queer, genderqueer, asexual, agender etc.). Studies assessing prevalence of depressive disorders were the primary focus of the SR identified; further research is needed to clarify prevalence rates of other mental disorders, which can then be synthesised in future SR. Furthermore, most studies synthesised by the SR focused upon SGM young people from Europe and North America: SGM young people from non-Western cultures may have differing prevalence rates due to facing unique legislative and context-dependent stressors [204]. The research field needs to both acknowledge this in their work and attempt to include these groups further. In terms of clinical relevance, future research and SR should examine cross-cultural differences in prevalence to encourage the development of appropriate mental health services that acknowledge the diversity of SGM young people’s lived experience. Additionally, an integrative framework that examines the combination of social identities in affecting one’s lived experience [205], should also be employed in future research and SR to achieve this.

Our findings suggest whilst the SR literature has had a variety of focuses, they have consistently reported a disproportionate prevalence and severity of mental disorders in SGM young people, particularly from depression. These disparities can be linked to the various risk factors indicated by our review. Whilst it is important to note most SGM young people do not experience mental disorders, our findings have important implications for clinical settings, research and policy. Firstly, clinicians should be vigilant for mental disorders in SGM young people and could for example consider putting screening measures in place, and where possible early intervention, for this group. SGM identity could be considered a risk factor for mental disorders in a sensitive and non-discriminatory way within groups of young people. Second, our findings have indicated the risk factors that have been focused upon within the review literature. This should provide stakeholders with a comprehensive foundation to develop potential pathways towards both reducing and preventing the disparities identified; for example, in the development of health interventions rooted in contextual factors [206, 207]. Third, our findings can inform researchers of the factors that have not been examined consistently within this field; further research into these understudied pathways for the development of mental disorders in this group of young people is needed. Research also needs to assess the additive effects of individual risk factors in mental health outcomes, given these risk factors are unlikely to exist in isolation. However, this research should use more representative sampling techniques including population level research. In addition, efforts should be made to collaborate with SGM young people directly in coproducing the services that will have the most benefit to this group.

## Electronic supplementary material

Below is the link to the electronic supplementary material.

Supplementary Material 1

Supplementary Material 2

Supplementary Material 3

Supplementary Material 4

## Data Availability

No datasets were generated or analysed during the current study.
