# Supplementary material for: Prevalence, severity and risk factors for mental disorders among sexual and gender minority young people: a systematic review of systematic reviews and meta-analyses
Source: Eur Child Adolesc Psychiatry. 2024 Aug 14;34(3):959–82. doi: 10.1007/s00787-024-02552-1 (PMC11909030; doi:10.1007/s00787-024-02552-1)
Supplement: Supplementary file 1 — Supplementary Material 1 [file 787_2024_2552_MOESM1_ESM.docx]

Table S1: list of search terms for each database.

| **Database** | **Search terms** |
| --- | --- |
| **MEDLINE** | 1. exp "Sexual and Gender Minorities"/ or exp Sexuality/ or exp Gender Identity/  2. (lgb* or sexual orientation or sexual identity or sexual preference or queer or gay or lesbian or bisexual or MSM or WSW or omnisexual or pansexual or asexual or aromantic or mostly gay or mostly lesbian or mostly homosexual or mostly heterosexual or trans or transgender or transsexual or gender nonconformi* or gender dysphoria or non-binary or genderqueer or gender fluid or intersex or AMAB or AFAB).mp. [mp=title, abstract, heading word, table of contents, key concepts, original title, tests & measures, mesh word]  3. 1 or 2  4. exp Mental Health/ or exp Mental Disorders/ or "Diagnostic and Statistical Manual of Mental Disorders"/  5. (mental health or mental disorder$ or mental illness or psychiatric disorder$ or psychological disorder$ or psychological distress).mp. [mp=title, abstract, heading word, table of contents, key concepts, original title, tests & measures, mesh word]  6. 4 or 5  7. risk factors/  8. incidence/ or prevalence/  9. (moderat* or mediat* or risk or mechan* or predict* or pathway or interact* or protective or causal or facto* or influence or correlate* or precurs* or prevalence or incidence or epidemiology or rate*).mp. [mp=title, abstract, heading word, table of contents, key concepts, original title, tests & measures, mesh word]  10. 7 or 8 or 9  11. adolescent psychiatry/ or child psychiatry/  12. (adolescen* or child or youth or young adult).mp. [mp=title, abstract, heading word, table of contents, key concepts, original title, tests & measures, mesh word]  13. 11 or 12  14. 3 and 6 and 10 and 13 |
| **PsycINFO** | 1. exp gender identity/ or exp sexual orientation/ or Sexual Minority Groups/  2. (lgb* or sexual orientation or sexual identity or sexual preference or queer or gay or lesbian or bisexual or MSM or WSW or omnisexual or pansexual or asexual or aromantic or mostly gay or mostly lesbian or mostly homosexual or mostly heterosexual or transgender or trans or transsexual or gender nonconformi* or gender dysphoria or non-binary or genderqueer or gender fluid or intersex or AMAB or AFAB).mp. [mp=title, abstract, heading word, table of contents, key concepts, original title, tests & measures, mesh word]  3. 1 or 2  4. exp Mental Disorders/ or exp Mental Health/ or exp "Diagnostic and Statistical Manual"/  5. (mental health or mental disorder$ or mental illness or psychiatric disorder$ or psychological disorder$ or psychological distress).mp. [mp=title, abstract, heading word, table of contents, key concepts, original title, tests & measures, mesh word]  6. 4 or 5  7. risk factors/  8. epidemiology/  9. (moderat* or mediat* or risk or mechan* or predict* or pathway or interact* or protective or causal or facto* or influence or correlate* or precurs* or prevalence or incidence or epidemiology or rate*).mp. [mp=title, abstract, heading word, table of contents, key concepts, original title, tests & measures, mesh word]  10. 7 or 8 or 9  11. exp Child Psychopathology/ or exp Child Psychiatry/ or exp Child Psychology/  12. exp Adolescent Psychiatry/ or exp Adolescent Psychology/ or exp Adolescent Psychopathology/  13. (adolescen* or child or youth or young adult).mp. [mp=title, abstract, heading word, table of contents, key concepts, original title, tests & measures, mesh word]  14. 11 or 12 or 13  15. 3 and 6 and 10 and 14 |
| **Scopus and Web of Science** | lgb* OR “sexual orientation” OR “sexual minorit*” OR “sexual identity” OR “sexual preference” OR queer OR gay OR lesbian OR bisexual OR msm OR wsw OR omnisexual OR pansexual OR asexual OR aromantic OR “mostly gay” OR “mostly lesbian” OR “mostly homosexual” OR “mostly heterosexual” OR transgender OR trans OR transsexual OR “gender nonconformi*” OR “gender minorit*” OR “gender dysphoria” OR “non-binary” OR genderqueer OR “gender fluid” OR intersex OR AMAB OR AFAB  AND  "mental health" OR “mental disorder$” OR “mental illness” OR “psychiatric disorder$” OR “psychological disorder$” OR “psychological distress”  AND  moderat* OR mediat* OR risk OR mechan* OR predict* OR pathway OR interact* OR protective OR causal OR facto* OR influence OR correlate* OR precurs* OR prevalence OR incidence OR epidemiology OR rate*  AND  adolescen* or child or youth or “young adult” |
