# Supplementary material for: Prevalence, severity and risk factors for mental disorders among sexual and gender minority young people: a systematic review of systematic reviews and meta-analyses
Source: Eur Child Adolesc Psychiatry. 2024 Aug 14;34(3):959–82. doi: 10.1007/s00787-024-02552-1 (PMC11909030; doi:10.1007/s00787-024-02552-1)
Supplement: Supplementary file 2 — Supplementary Material 2 [file 787_2024_2552_MOESM2_ESM.docx]

## Table S2: NOS scoring.

|  | Representativeness of the sample | Sample Size | Characteristics of non-respondents | Ascertainment of the exposure | Comparability of groups | Assessment of the outcome | Statistical test | Overall quality |
| --- | --- | --- | --- | --- | --- | --- | --- | --- |
| Williams & Chapman (2011) | * | * |  | * | ** | * | * | High |
| Lucassen et al. (2015) | * | * |  | * | ** | * | * | High |
| Noell & Ochs (2001) |  | * | * | * | ** | ** | * | High |
| Denny et al. (2014) | * | * |  | * | ** | * | * | High |
| Mustanski et al. (2010) | * | * |  | * |  | ** | * | Moderate |
| Rosario et al. (2014) | * | * | * | * | ** | * | * | High |
| Wichstrøm (2006) | * | * | * | * | ** | * | * | High |
| Whitbeck et al. (2004) |  | * |  | * | ** | ** | * | High |
| Lea et al. (2014) | * | * | * | * |  | * | * | Moderate |
| Burns et al. (2015) | * | * |  | * |  | ** | * | Moderate |
| Luk et al. (2018) | * | * |  | * | ** | * | * | High |
| Becker et al. (2014) | * | * |  | * | ** | * | * | High |
| Rivers & Noret (2008) | * | * |  | * | ** | * | * | High |
| Williams et al. (2005) | * | * |  | * | ** | * | * | High |
| Pesola et al. (2014) | * | * | * | * | ** | * | * | High |
| Martin-Storey & Crosnoe (2012) | * | * |  | * | ** | * | * | High |
| Safren & Heimberg (1999) |  | * |  | * | ** | * | * | Moderate |
| Gattis (2013) |  | * |  | * | ** | * | * | Moderate |
| Hatzenbuehler et al. (2008) | * | * |  | * | ** | * | * | High |
| Lam et al. (2004) | * | * |  | * | ** | * | * | High |
| Irish et al. (2018) | * | * |  | * | ** | * | * | High |
| Cheng et al. (2016) | * | * |  | * | ** | * | * | High |
| Ueno (2010) | * | * |  | * | ** | * | * | High |
| Johnson et al. (2011) | * | * | * | * | ** | * | * | High |
| Marshal et al. (2012) | * | * | * | * | ** | * | * | High |
| Marshal et al. (2013) | * | * |  | * | ** | * | * | High |
| Ziyadeh et al. (2007) | * | * |  | * | ** | * | * | High |
| Galliher et al. (2004) | * | * |  | * | ** | * | * | High |
| Gisladottir et al. (2018) | * | * |  | ** | ** | * | * | High |
| Pachankis & Goldfried (2006) | * | * |  | * | ** | * | * | High |
| Austin et al. (2004) | * | * |  | * | ** | * | * | High |
| Jorm et al. (2002) | * | * |  | * | ** | * | * | High |
| de Vries et al. (2011) |  | * | * | ** |  | * | * | Moderate |
| Clark et al. (2014) | * | * |  | * | ** | * | * | High |
| Katz-Wise et al. (2018) | * |  |  | * |  | * | * | Moderate |
| Russell et al. (2022) | * | * |  | * | ** | * | * | High |
| Price-Feeney et al. (2018) | * | * |  | * | ** | * | * | High |
| Moyer et al. (2019) |  | * | * | ** |  | * | * | Moderate |
| Durwood et al. (2017) | * | * |  | * | ** | * | * | High |
| Eisenberg et al. (2017) |  |  |  | * | ** | * | * | High |
| Laggari et al. (2009) |  |  |  | ** | ** | * | * | Moderate |
| Veale et al. (2017) | * | * |  | * |  | ** | * | Moderate |
| Wallien et al. (2007) |  | * |  | ** | ** | ** | * | High |
| VanderLaan et al. (2018) |  | * |  | ** |  | * | * | Moderate |
| Olson et al. (2016) | * | * |  | * | ** | * | * | High |
| Wang et al. (2020) | * | * |  | * | ** | * | * | High |
| Mustanski & Liu (2013) | * | * |  | * | ** | ** | * | High |
| Parodi et al. (2022) | * | * |  | * |  | * | * | Moderate |
| Kozlowska et al. (2021) |  |  |  | ** |  | * | * | Low |
| Tordoff et al. (2022) |  | * |  | * |  | * | * | Low |
| Perales & Campbell (2019) | * | * |  | * | ** | * | * | High |
