# Supplementary material for: Prevalence, severity and risk factors for mental disorders among sexual and gender minority young people: a systematic review of systematic reviews and meta-analyses
Source: Eur Child Adolesc Psychiatry. 2024 Aug 14;34(3):959–82. doi: 10.1007/s00787-024-02552-1 (PMC11909030; doi:10.1007/s00787-024-02552-1)
Supplement: Supplementary file 3 — Supplementary Material 3 [file 787_2024_2552_MOESM3_ESM.docx]

## Table S3: Means (SD) among SM and heterosexual young people identified in studies not meta-analysed.

| **Study** | **Assessment method** | **SM M (SD)** | **Heterosexual M (SD)** | ***p*** |
| --- | --- | --- | --- | --- |
| **Depressive Disorders** | | | | |
| Rosario et al. (2014) | McKnight Risk Factor Survey | Lesbian/gay = 6.9 (3.7)  Bisexual = 7.7 (4.4)  Mostly heterosexual = 7.2 (3.7) | 5.9 (3.3) | Lesbian/gay vs. heterosexual < .01  Bisexual vs. heterosexual < .001  Mostly heterosexual vs. heterosexual < .001 |
| Gisladottir et al. (2018) | Symptom Checklist 90 | Lesbian/gay = 10.04 (7.74)  Bisexual = 13.29 (8.35) | 8.07 (6.9) | .02 |
| Galliher et al. (2004) | Adapted version of Center for Epidemiological Studies Depression Scale | Lesbian from rural area = 0.69 (0.54)  Bisexual female from rural area = 0.66 (0.65)  Gay male from rural area = 0.40 (0.45)  Bisexual male from rural area = 0.40 (0.34)  Lesbian from urban area = 0.34 (0.33)  Bisexual female from urban area = 0.54 (0.48)  Gay male from urban area = 0.69 (0.67)  Bisexual male from urban area = 0.42 (0.40)  Lesbian from suburban area = 0.54 (0.56)  Bisexual female from suburban area = 0.66 (0.58)  Gay male from rural area = 0.39 (0.40)  Bisexual male from rural area = 0.45 (0.47) | Girls from rural area = 0.45 (0.45)  Girls from urban area = 0.47 (0.47)  Girls from suburban area = 0.44 (0.45)  Boys from rural area = 0.28 (0.35)  Boys from urban area = 0.33 (0.35)  Boys from suburban area = 0.32 (0.38) | Lesbian/gay vs. heterosexual = .004  Bisexual vs. heterosexual < .001 |
| Ziyadeh et al. (2007) | McKnight Risk Factor Survey | Mostly heterosexual males = 1.3  Gay/bisexual males = 1.2  Questioning/unsure males = 1.2  Mostly heterosexual females = 1.6  Lesbian/bisexual females = 1.6  Questioning/unsure females = 1.3  *SD/SE not reported.* | Males = 1.1  Females = 1.2 | Mostly heterosexual vs. heterosexual males < .001  Gay/bisexual vs. heterosexual males not significant  Questioning/unsure vs. heterosexual males < .05  Mostly heterosexual vs. heterosexual females < .001  Lesbian/bisexual vs. heterosexual females < .01  Questioning/unsure vs. heterosexual females < .05 |
| **Anxiety Disorders** | | | | |
| Hatzenbuehler et al. (2008) | Multidimensional Anxiety Scale for Children | 46 (14.42) | 40.03 (15.4) | < .05 |
| Marshal et al. (2012) | Screen for Child Related Emotional Disorders | 19.4 (12.1) | 15.5 (9.5) | < .05 |
| Marshal et al. (2013) | Screen for Child Related Emotional Disorders | 15.1 (10.4) | 13.43 (9.44) | Not reported |
| Pachankis & Goldfried (2006) | Social Interaction Anxiety Scale | 25.18 (12.58) | 18.71 (10.86) | < .001 |
|  | Social Phobia Scale | 18.53 (11.82) | 15.56 (9.35) | Not significant |
|  | Fear of Negative Evaluation Scale | 16.24 (8.2) | 11.75 (6.92) | < .001 |
|  | Inventory of Anxiousness | 2.8 | 1.86 | < .001 |
| **Oppositional Defiant Disorder** | | | | |
| Marshal et al. (2012) | Child Symptom Inventory | 7.1 (4.2) | 5.1 (3.7) | < .01 |
| Marshal et al. (2013) | Adolescent/Adult Self-Report Inventory-4 | 0.83 (0.52) | 0.83 (0.53) | Not reported |
| **Conduct Disorder** | | | | |
| Marshal et al. (2012) | Child Symptom Inventory | 2.2 (3.0) | 1.3 (2.0) | < .05 |
| Marshal et al. (2013) | Adolescent/Adult Self-Report Inventory-4 | 0.15 (0.19) | 0.08 (0.11) | Not reported |
| **Borderline Personality Disorder/Emotionally Unstable Personality Disorder** | | | | |
| Marshal et al. (2012) | International Personality Disorders Examination | 4.2 (2.8) | 2.8 (2.2) | < .01 |
| Marshal et al. (2013) | International Personality Disorders Examination | 3.28 (2.10) | 2.19 (1.79) | Not reported |
| **Tobacco Use Disorder** | | | | |
| Austin et al. (2004) | Stanford Dependence Index | Mostly heterosexual girls = 9.2  Lesbian/bisexual girls = 11.5  Mostly heterosexual boys = 8.5  Gay/bisexual boys = 8.2  SD/SE not reported | Girls = 8.1  Boys = 8.3 | Not reported |
| **Alcohol Use Disorder** | | | | |
| Pesola et al. (2014) | Alcohol Use Disorders Identification Test | 7.9 (5.5) | 6.6 (4.8) | Not reported |
