# Supplementary material for: Prevalence, severity and risk factors for mental disorders among sexual and gender minority young people: a systematic review of systematic reviews and meta-analyses
Source: Eur Child Adolesc Psychiatry. 2024 Aug 14;34(3):959–82. doi: 10.1007/s00787-024-02552-1 (PMC11909030; doi:10.1007/s00787-024-02552-1)
Supplement: Supplementary file 4 — Supplementary Material 4 [file 787_2024_2552_MOESM4_ESM.docx]

## Table S4: Means (SD) among GM and cisgender young people identified in studies not meta-analysed.

| **Study** | **Assessment method** | **Trans* M (SD/SE)** | **Cisgender M (SD/SE)** | ***p*** |
| --- | --- | --- | --- | --- |
| **Depressive Disorders** | | | | |
| Russell et al. (2022) | Child Behaviour Checklist | 57.3 (1.27) | 53.7 (0.08) | Not reported |
| Durwood et al. (2017) | Patient-Reported Outcomes Measurement Information System | 48.7 (9.4) | Matched controls = 46.4 (8.0)  Siblings = 47.9 (7.9) | Not significant |
| Olson et al. (2016) | Patient-Reported Outcomes Measurement Information System | 50.1^✝^ | Control = 48.4^✝^  Sibling = 49.3^✝^ | Not significant |
| Wang et al. (2020) | Patient Health Questionnaire-9 | Trans males = 8.28 (7.35)  Trans females = 8.03 (7.78)  Non-binary AMAB M = 7.78, SD = 7.95  NB AFAB M = 8.45, SD = 7.46;  Questioning AMAB M = 7.81, SD = 7.27;  Questioning AFAB M = 7.65, SD = 6.49 | Cisgender males = 4.63 (5.63)  Cisgender females = 5.13 (5.83) | Not reported |
| Mustanski & Liu (2013) | Diagnostic Interview Schedule for Children (DISC) | 10.43 (4.77) | Males = 9.69 (4.18)  Females = 10.09 (4.47) | Not significant |
| Laggari et al. (2009) | Beck Depression Inventory | 19.0 (10.93) | 10.32 (7.19) | Not reported |
| **Anxiety Disorders** | | | | |
| Russell et al. (2022) | Child Behaviour Checklist | 57 (1.25) | 53.4 (0.08) | Not significant |
| Durwood et al. (2017) | Patient-Reported Outcomes Measurement Information System | 52 (9.6) | Matched controls = 49 (7.7)  Siblings = 52.8 (10.5) | Not significant |
| Olson et al. (2016) | Patient-Reported Outcomes Measurement Information System | 54.2 | Control = 50.9  Sibling = 52.3 | Not significant |
| Wang et al. (2020) | Generalized Anxiety Disorder 7-item | Trans males M = 7.26, (5.81)  Trans females = 7.0 (6.3)  Non-binary AMAB = 7.01 (6.78)  Non-binary AFAB = 7.51 (6.01)  Questioning AMAB = 6.85 (6.05)  Questioning AFAB = 6.74 (5.22) | Cisgender males = 4.43 (4.72)  Cisgender females = 5.33 (4.87) | Not reported |
| Laggari et al. (2009) | State-Trait Anxiety Inventory | 36.55 (10.44) | 31.5 (8.24) | Not reported |
| **Conduct Disorder** | | | | |
| Russell et al. (2022) | Child Behaviour Checklist | 56.4 (1.52)^✝^ | 53.2 (0.08)^✝^ | Not reported |
| **Somatic Symptom Disorder** | | | | |
| Russell et al. (2022) | Child Behaviour Checklist | 57.0 (1.25)^✝^ | 53.4 (0.08)^✝^ | Not reported |

^✝^ mean t-scores reported
